# Idiopathic fibrosing mediastinitis

**DOI:** 10.7196/AJTCCM.2021.v27i2.064

**Published:** 2021-06-23

**Authors:** F D Manyeruke, R Perumal, G Symons, L Mottay

**Affiliations:** Division of Pulmonology, Department of Medicine, Faculty of Health Sciences, University of Cape Town and Groote Schuur Hospital, Cape Town, South Africa

**Keywords:** idiopathic fibrosing mediastinitis, fibrosing mediastinitis, haemoptysis

## Abstract

Fibrosing mediastinitis is rare in settings where histoplasmosis is not endemic. An idiopathic form of the disease may present with indistinguishable features and requires methodical exclusion of competing differential diagnoses. We report the case of a 30-year old female patient who presented with intermittent haemoptysis for the past 2 years with no constitutional symptoms. Computed tomography of the chest revealed a prominent right bronchial arterial circulation with a mass-like lesion, which encased and attenuated the right pulmonary trunk and adjacent structures. Endobronchial ultrasonography with transbronchial fine-needle aspiration showed a paucicellular aspirate with no evidence of malignancy or granulomas. Fungal infection, tuberculosis, sarcoidosis, IgG4-disease, and connective tissue disease were ruled out by appropriate serological, molecular, and microbiological tests. A diagnosis of idiopathic fibrosing mediastinitis was therefore made by exclusion and the patient was successfully treated with oral corticosteroids.

## Background

Fibrosing mediastinitis is a clinical pathological syndrome rather than a single disease entity. Fibrosing mediastinitis has been associated with a number of triggers with variable geographic epidemiology.^[1]^ These include infections like histoplasmosis, tuberculosis, aspergillosis, blastomycosis and cryptococcosis. Histoplasmosis is the most common cause of fibrosing mediastinitis in North America,^[2]^ accounting for up to 78% of cases. Fibrosing mediastinitis has also been associated with autoimmune diseases, radiation therapy, Hodgkin disease, rheumatic fever, trauma, drugs, IgG4-related disease and Behçet’s disease. In a region where histoplasmosis is not endemic, idiopathic fibrosing mediastinitis accounts up to 58% of all cases of fibrosing mediastinitis.^[3]^ There is a paucity of data on fibrosing mediastinitis in sub-Saharan Africa. We present our experience in managing a patient with this rare disease.

## Case

A 30-year-old female patient presented to the clinic with intermittent haemoptysis for the past 2 years. She was a lifetime non-smoker, had no significant organic or inorganic dust exposures, and had not recently travelled. She reported progressively worsening dyspnoea and a chronic non-productive cough. There were no constitutional symptoms or a history of tuberculosis.

A review of her past medical history revealed that 2 years prior to consulting us, she presented to a different pulmonology unit following an episode of massive haemoptysis. Following extensive investigations, the only identifiable pathology was that of an enlarged right hilar lymph node. A biopsy of the lymph node was performed via a thoracotomy and revealed a hyalinising granuloma. The patient was lost to follow-up until her presentation to our unit.

**Fig. 1:**
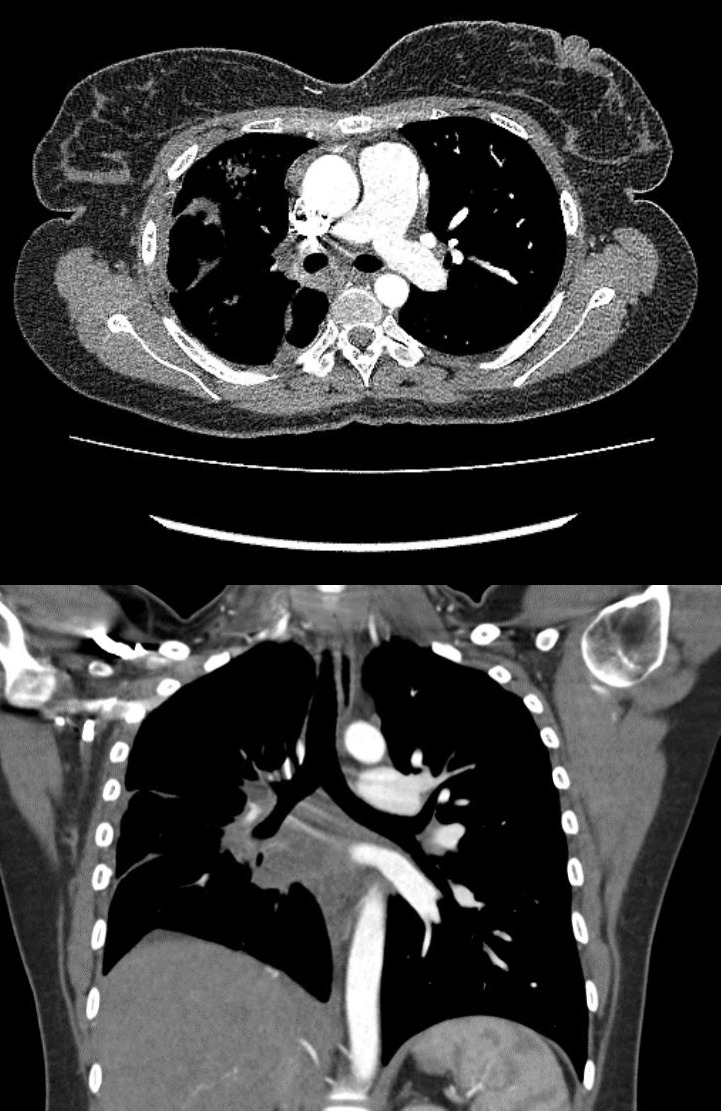
Computed tomography scans showing a mass extending and involving the right hilar and subcarinal region, encasing the right main bronchus and right pulmonary trunk.

No abnormal signs were elicited on physical examination. In particular, we found no clinical signs suggestive of tuberculosis, sarcoidosis, or connective tissue disease. Spirometry showed mild restriction and a reduced diffusing capacity of the lung for carbon monoxide. A chest radiograph showed reduced volume of the right hemithorax and a right hilar mass-like opacity. Computed tomography found a prominent right bronchial arterial circulation with a distended bronchial artery, tortuous fifth to seventh intercostal arteries, and collateralisation of the pulmonary circulation. A right hilar soft tissue mass was found to be encasing the right pulmonary artery with resultant attenuation of the vessel (Fig. 1). Multifocal areas of ground-glass opacification were seen in the right upper and middle lobes with associated interlobular septal thickening consistent with pulmonary haemorrhage. The previous diagnosis of hyalinising granuloma was excluded after review of the radiology.

Microbiological examination of expectorated sputum was negative for tuberculosis, and cytological evaluation was unrevealing. Serological studies for histoplasmosis, *Aspergillus* and connective tissue disease were negative. Serum angiotensin-converting enzyme, IgG4 and calcium were within normal limits.

We proceeded to perform an endobronchial ultrasound with transbronchial fine-needle aspiration of the subcarinal component of the mass-like lesion. No endobronchial lesions were observed during the bronchoscopy, and all major and segmental airways were patent. The aspirate was evaluated by microscopic cytopathological examination, flow cytometry, mycology and mycobacterial culture. Cytopathological examination revealed a paucicellular aspirate with no evidence of malignancy or granulomatous disease, flow cytometry was normal, and microbiological culture examination was negative for tuberculosis and there was no growth on mycology culture.

A diagnosis of idiopathic fibrosing mediastinitis was made following consensus by a multi-disciplinary team, and on the basis of highly suggestive radiology (a constrictive, fibrosing lesion of the right hilum and adjacent mediastinal structures) and the exclusion of competing differential diagnoses. Treatment with high-dose oral corticosteroids was initiated and the patient remains clinically stable without evidence of disease progression during follow-up care. She has had no further episodes of haemoptysis.

## Discussion

**Fig. 2:**
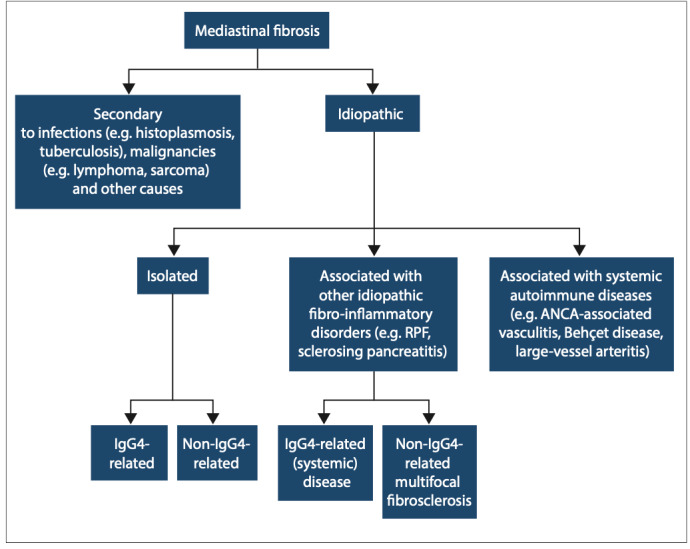
Classification of mediastinal fibrosis. RPF = retroperitoneal fibrosis ANCA = antineutrophil cytoplasmic antibodies

Idiopathic fibrosing mediastinitis is a metabolically active space-occupying fibro-inflammatory disease in the mediastinum.^[4]^ Many hypotheses have been advanced for its aetiology; however, the exact cause remains unknown. IgG4-related disease has been recognised as having significant overlap with idiopathic fibrosing mediastinitis and must be actively excluded with use of serum levels of IgG4 and histological markers.^[4,5]^ Idiopathic fibrosing mediastinitis has also been associated with other idiopathic fibro-inflammatory disorders and autoimmune disease (Fig. 2).^[6]^

The microscopic characteristics of fibrosing mediastinitis reveal abundant, paucicellular, fibrous tissue infiltrating and obstructing adipose tissue. Granulomas are usually absent in patients with idiopathic fibrosing mediastinitis. Idiopathic fibrosing mediastinitis is progressive when left untreated and can be staged as follows:

- stage 1 lesions are characterised by oedematous fibromyxoid tissue associated with an inflammatory reaction, thin-walled vessels, and lacking cellular atypia and necrosis.
- stage 2 lesions are poorly demarcated lesions consisting of haphazardly arranged hyaline material encircling and infiltrating mediastinal structures with minimal inflammatory reaction.
- stage 3 lesions are obliterative, which involve mediastinal structures, and are characterised by acellular dense collagen and occasional lymphoid aggregates. Spindle cells and inflammatory cells are absent. Dystrophic calcification is commonly seen.^[4,7]^

Idiopathic fibrosing mediastinitis may present with one of two main radiological patterns – focal or diffuse disease. Focal fibrosing mediastinitis usually involves the right mediastinum, hilar and subcarinal region.^[3]^ The diffuse type can extend to the soft-tissue structures of the neck, posterior mediastinum and the lung. The majority of patients will experience symptoms and exhibit signs of compression of mediastinal structures, which include pulmonary arterial or venous narrowing, superior vena cava obstruction, and airway narrowing.^[8]^

Affected patients are usually young and present with symptoms related to obstruction of vital mediastinal structures, such as the oesophagus, airways, pulmonary arteries or veins and central systemic veins. The most common presenting complaints include cough, dyspnoea, haemoptysis and pleuritic chest pain.^[9]^ Haemoptysis can affect up to 20% of patients and has several potential causes in patients with fibrosing mediastinitis.^[1]^ Airway obstruction with a post-obstructive necrotising pneumonia, invasion of a bronchus by fibrous tissue, and pulmonary hypertension from pulmonary vascular compression are the most common clinically encountered mechanisms. Obstruction of the pulmonary arteries, in particular, may lead to extensive anastomoses with intercostal or bronchial arteries, which increases the risk for massive haemoptysis.^[10]^

Idiopathic fibrosing mediastinitis is usually a progressive disease with no evidence-based therapeutic options. Oral corticosteroids are the most commonly used medical treatment and have been associated with variable success. Other potentially efficacious therapies include tamoxifen, methotrexate and mycophenolate mofetil.^[11–14]^ Rituximab has also been shown to be associated with a favourable therapeutic response in patients with progressive and refractory disease.^[15]^ Surgical biopsy should be performed in patients with poor response to therapy. Surgical resection is curative in localised disease and may ameliorate symptoms. A complete resection may require vascular and airway reconstruction, which is associated with high morbidity and mortality. Patients with bilateral mediastinal involvement, extensive fibrosis, calcifications and collateral vessels are generally not suitable for surgery.^[16]^ Symptomatic patients can also be treated with local therapies directed towards re-establishing patency of occluded airways, pulmonary arteries or vena cava.^[17]^

## Conclusion

This case highlights a common presentation of a rare disease and the challenge of establishing the diagnosis of idiopathic fibrosing mediastinitis. The diagnosis of idiopathic fibrosing mediastinitis requires suggestive radiological and pathological findings, and the methodical exclusion of competing differential diagnoses.
